# Metabolic effects of an aspartate aminotransferase-inhibitor on two T-cell lines

**DOI:** 10.1371/journal.pone.0208025

**Published:** 2018-12-07

**Authors:** Henrik Antti, Magnus Sellstedt

**Affiliations:** Department of Chemistry, Umeå University, Umeå, Sweden; University of South Alabama Mitchell Cancer Institute, UNITED STATES

## Abstract

An emerging method to help elucidate the mode of action of experimental drugs is to use untargeted metabolomics of cell-systems. The interpretations of such screens are however complex and more examples with inhibitors of known targets are needed. Here two T-cell lines were treated with an inhibitor of aspartate aminotransferase and analyzed with untargeted GC-MS. The interpretation of the data was enhanced by the use of two different cell-lines and supports aspartate aminotransferase as a target. In addition, the data suggest an unexpected off-target effect on glutamate decarboxylase. The results exemplify the potency of metabolomics to provide insight into both mode of action and off-target effects of drug candidates.

## Introduction

Determination of the mode of action of novel drug candidates is an important part of the drug discovery process and typically requires a multitude of experimental techniques–from genetic methods to proteomics and chemical biological methods such as pull-down experiments with resin-bound drug analogs.[1] A less frequently used method is untargeted metabolomics screens.[2] The method has mainly been used for determination of the mode of action of antimicrobial agents, which in many cases have proven very successful–especially for enzymatic targets.[3–10] The use of cell-lines to probe the mode of action of experimental drugs is less common, although there are successful examples.[11–18] Untargeted metabolomics screens are rather cost- and labor-effective and can give important and complementary information to other methods in the elucidation of a compounds biological mode of action. However, the interpretation of the metabolomics data from such screens can be difficult. To get a better understanding of how small molecules can affect the metabolic profile of cell-lines, more studies of compounds with known targets are still needed. Suspension cells are convenient to use in metabolomics studies as they can be maintained at high cell densities. In addition, their mode of growth more closely resembles their normal counterparts compared to adherent 2D-cell cultures. Molt-16 and Jurkat E6.1 are useful suspension cells models derived from T-cell leukemias. Here we describe how an aspartate aminotransferase inhibitor alters the metabolome in these two T-cell lines.

**Fig 1:**
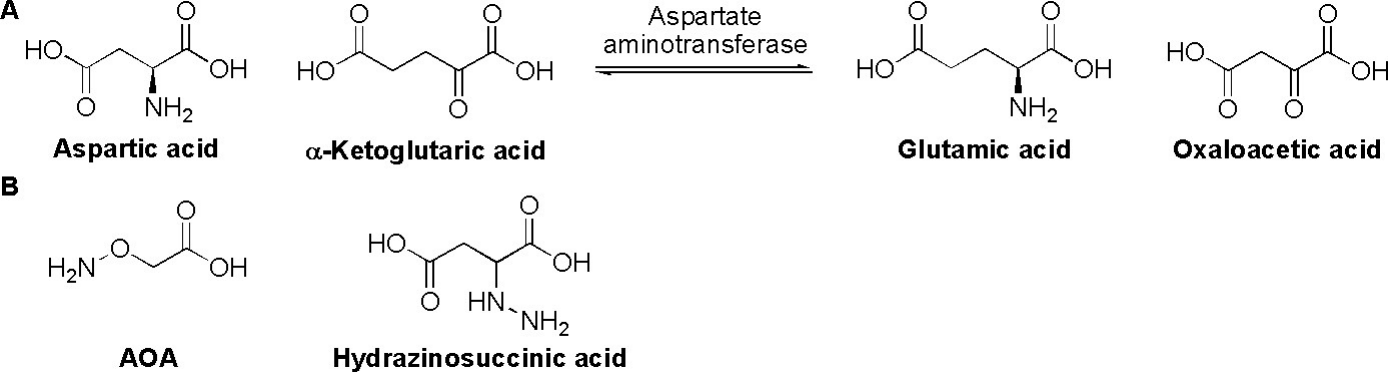
**A)** AAT catalysis. **B)** Inhibitors of AAT.

Aspartate aminotransferase (AAT) catalyze the reversible interchange of aspartate and α-ketoglutaric acid to glutamic acid and oxaloacetic acid (Fig 1A). The enzyme has been suggested as a target to selectively kill breast-cancer cells over normal mammalian tissue, and the effects of the inhibitor aminooxyacetic acid (AOA) (Fig 1B) have previously been studied [19]. As all aminotransferases AAT requires binding of pyridoxal phosphate for activity. AOA inactivates pyridoxal phosphate-bound aminotransferases by reacting with the aldimine bond between these enzyme components. AOA is, however, only a moderately potent inhibitor of AAT with typical reported IC_50_ values in excess of 100 μM. A more potent inhibitor is hydrazinosuccinic acid (Fig 1B), which has similar inhibition mechanism as AOA but two orders of magnitude lower K_i_ value [20]. The improved structural similarity between hydrazinosuccinic acid and the enzyme’s natural substrates compared to AOA is also likely to give more selective inhibition of AAT over other pyridoxal phosphate binding enzymes. Hence, hydrazinosuccinic acid was chosen as inhibitor to study the metabolic effects of AAT inhibition.

## Materials and methods

### Synthesis of hydrazinosuccinic acid hydrate

Maleic acid, 87 mg (0.75 mmol), and *tert*-butyl carbazate, 104 mg (0.79 mmol) was taken up in 0.6 mL water and heated in a sealed tube at 37 °C for 6 days. The reaction was cooled to room temperature and then 0.24 mL concentrated HCl(aq.) was added. After stirring for 2.5 h at room temperature the reaction was concentrated under reduced pressure and purified with reversed phase HPLC (C18) using water:acetonitrile+0.75% formic acid as mobile phase and 5–20% acetonitrile over 20 min. as gradient at a flow rate of 20 mL/min. The product was collected in fraction 3–5 and detected on TLC using potassium permanganate staining. 40 mg (32%) of a white solid was obtained. NMR-data was in agreement with previously published data [20].

### Preparation of cell samples

Molt-16 cells (DSMZ ACC 29) or Jurkat E6.1 cells (ATCC TIB-152) were cultured in RPMI 1640 media + 10% heat inactivated FBS + 50 U/mL Penicillin-streptomycin.

#### Typical procedure

6-Welled plates were charged with 6 mL / well of suspensions of cells at a density of approximately 4.5–7.5 * 10^5^ live cells / mL. PBS, 6 μL (controls), or 10 mM hydrazinosuccinic acid in PBS, 6 μL, was added to each well and the plates were incubated at 37 °C and 5% CO_2_ for 48 h. The cells were suspended by repeated pipetting and the cell density was determined using 10 μL counting chambers. The cell suspensions were then transferred to 15 mL centrifuge tubes and centrifuged at 400 g for 4 min at 4 °C. Working on ice, the supernatants were discarded and the cell pellets were resuspended in 2 mL cold PBS and centrifuged at 400 g for 4 min at 4 °C. The supernatants were again discarded and the cell pellets were washed twice more with PBS in this manner before the pellets were stored at -80 °C.

### Sample extraction

The cell pellets were thawed on ice and then transferred to Eppendorf tubes using 500 μL extraction mixture (methanol:water 9:1 containing internal standards). A tungsten bead was added to each tube and the samples were extracted using a vibration mill for 2 min at 30 Hz. The samples were stored for 30 min at 0 °C and the tungsten beads were then removed and the samples were centrifuged at 14000 rpm (18620 g) for 10 min at 4 °C. 300 μL supernatant solutions from each sample were transferred to GC-vials and concentrated to complete dryness under reduced pressure at room temperature and then stored at -80 °C.

### Derivatization of samples

Before GC-MS analysis the samples were derivatized. The samples were thawed and then concentrated under reduced pressure at room temperature for 20 min to remove any condensed moisture. 30 μL of a 15 μg/μL methoxamine solution in pyridine was added and the samples were dissolved by shaking for 10 min and then allowed to stand for 20 h at room temperature. Then 35 μL of a 6:1 mixture of MSTFA with 1% TMSCl and a solution of methyl stearate in heptane (100 ng / μL heptane) was then added and briefly vortexed and allowed to stand at room temperature for 1 h.

### GC analysis

GC-MS analysis was performed on a Leco Pegasus HT TOF-MS equipped with an Agilent 7890A gas chromatograph at the Swedish Metabolomics Center, Umeå, Sweden, https://www.swedishmetabolomicscentre.se/. The derivatized samples, 1 μl, were auto-injected (splittless) at a temperature of 270 °C. The ion source temperature was 200 °C and the ionization energy was 70 eV. Mass spectra were collected in the mass range 50 to 800 m/z. n-alkanes (C8-C40) were used as external retention index standards.

*For samples J1_1–6* a 30 m GC-column with an inner diameter of 0.25 mm was used. The purge delay time was 75 seconds and the rate was 20 mL/min. Helium was used as carrier gas (1 mL/min). The GC oven temperature was 70 °C for 2 minutes and then increased 20 °C/minute to 320 °C, where it was held constant for 8 minutes. The detector voltage was 1670 V.

*All other samples* employed a 10 m GC-column with an inner diameter of 0.18 mm. The purge delay time was 60 seconds and the rate was 20 mL/min. Helium was used as carrier gas (1 mL/min). The GC oven temperature was 70 °C for 2 minutes and then increased 40 °C/minute to 320 °C, where it was held constant for 2 minutes. The detector voltage was 1920 V.

### Data processing

The raw GC-MS data was aligned against the internal standards retention indexes and compared against an in-house spectral library of metabolites (Swedish Metabolomics Centre, Umeå, Sweden) using the in-house RDA software. The tentatively assigned metabolite data was curated using NIST MS search v2.0 and the annotated integrated data was normalized against the cell number and methyl stearate in each sample. For statistical evaluation univariate student’s t-tests were performed in Microsoft Excel and multivariate partial least squares-discriminant analysis (PLS-DA) was performed in SIMCA 14.0 (Umetrics AB, Umeå, Sweden).

## Results and discussion

Two different T-cell lines, Jurkat E6.1 and Molt-16, were treated with 10 μM of hydrazinosuccinic acid for 48 h and compared to untreated cells grown in parallel in triplicates. In addition, two independent replicates of treated and untreated Jurkat cells were included as a verification experiment. Using untargeted GC-MS analysis, 87 metabolites were identified in total (75 detected in all samples). The only metabolite showing significant change in all three experiment series was aspartic acid–one of the substrates of the target protein (Table 1). In addition, α-ketoglutaric acid, another substrate of AAT also scored high.

**Table 1:** Metabolites with at least one set of measurements with p < 0.1. The ratios of the detected metabolite levels in samples treated with 10 μM hydrazinosuccinic acid versus untreated controls are given. The data is sorted on log_2_ average ratios and colorized from red (metabolites increased in treated samples) to blue (metabolites decreased in treated samples). Measurements with p-values < 0.05 are highlighted in bold.

	Average		Molt-16, treated vs. untreated cells		Jurkat, treated vs. untreated cells		Jurkat, independent duplicate, treated vs. untreated cells	
	Log_2_-fold change	p-value	Ratio	p-value	Ratio	p-value	Ratio	p-value
Ribose	1.051	0.146	3.222	**0.033**	1.667	0.069	1.328	0.336
Aspartic acid	0.751	**0.018**	1.622	**0.012**	1.185	**0.035**	2.241	**0.007**
Ribose-5-phosphate	0.693	0.244	2.130	**0.008**	1.640	**0.007**	1.079	0.716
Nicotinamide	0.270	0.302	1.388	0.081	1.099	0.460	1.129	0.365
Glycerol-2-phosphate	0.260	0.272	1.565	**0.036**	0.916	0.227	1.111	0.552
Ribitol / other pentol	0.249	0.370	1.524	**0.041**	0.915	0.524	1.126	0.545
Sedoheptulose-7-phosphate	0.152	0.471	1.159	0.544	1.243	0.098	0.933	0.771
Fructose / sorbose	0.100	0.567	1.275	0.060	1.004	0.971	0.936	0.671
Glycerol-3-phosphate	0.096	0.449	1.283	**0.019**	0.925	0.339	0.999	0.988
Leucine	0.089	0.609	1.245	0.079	0.999	0.994	0.947	0.752
Valine	0.058	0.308	1.200	0.096	1.076	0.642	0.847	0.185
Tyrosine	0.052	0.343	1.166	0.083	1.059	0.560	0.884	0.387
Argininosuccinate derivative	0.040	0.243	1.238	**0.024**	0.774	0.073	1.072	0.634
Isoleucine	-0.009	0.336	1.165	0.099	0.986	0.896	0.831	**0.012**
Hypotaurine	-0.032	0.245	1.164	0.211	0.923	0.453	0.848	0.069
UDP-*N*-Ac-Glucosamine	-0.083	0.276	1.057	0.558	0.881	0.196	0.895	0.074
Aspargine	-0.093	0.407	1.058	0.495	0.816	**0.040**	0.939	0.688
Arginine	-0.115	0.422	1.081	0.430	0.979	0.777	0.710	0.059
Pyroglutamic acid	-0.162	0.408	1.042	0.669	0.956	0.519	0.683	**0.035**
Succinic acid	-0.167	0.412	0.977	0.809	1.096	0.395	0.599	**0.033**
Glutamic acid	-0.178	0.361	1.097	0.314	0.966	0.713	0.588	0.056
Citric acid / Isocitric acid	-0.188	0.363	1.027	0.744	0.931	0.327	0.675	**0.019**
4-Hydroxyproline	-0.206	0.389	0.995	0.961	0.865	0.166	0.740	**0.039**
Hypoxanthine	-0.232	0.604	0.929	0.771	0.654	0.075	0.972	0.966
Alanine	-0.368	0.173	0.700	0.082	0.823	0.213	0.802	0.224
Glycine	-0.469	0.244	0.848	0.440	0.931	0.273	0.389	**0.020**
α-Ketoglutaric acid	-0.592	0.132	0.754	**0.009**	0.664	**0.028**	0.572	0.361
4-Aminobutyric acid	-0.732	**0.049**	0.765	0.132	0.339	**0.0002**	0.702	**0.014**

**Fig 2:**
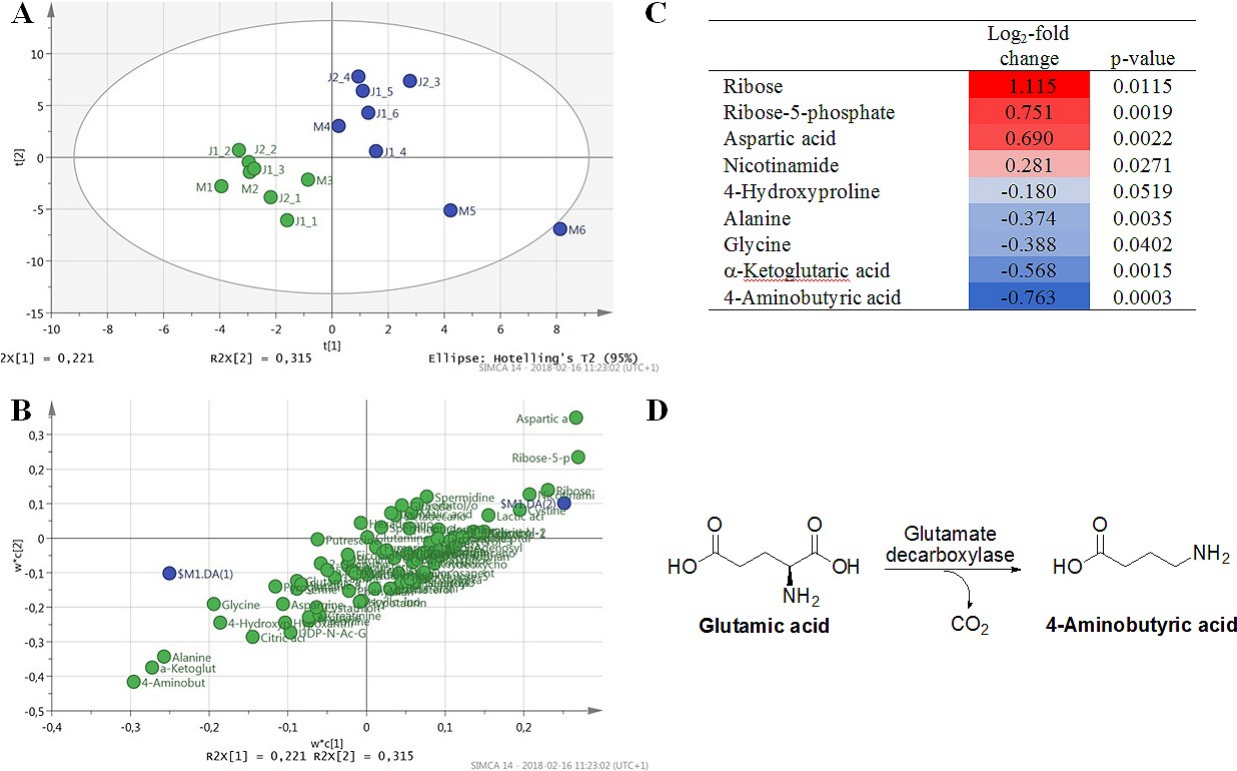
PLS-DA of the pooled data, mean centered and scaled to unit variance; 3 significant components, explained variation in metabolite data R2X = 0.640, explained between class variation R2Y = 0.989, predicted between class variation according to cross-validation Q2 = 0.886 A) Scores plot of the two first components (t2/t1) shows a separation between controls (blue) and treated samples (green). B) Corresponding overview of the two first components (w*c[2]/w*c[1]) showing an overview of the contribution of each metabolite (green) to the model in relation to the two sample classes; controls (DA(1), upper right quadrant) and treated samples (DA(2), lower left quadrant). C) List of all metabolites with p < 0.1 (student’s two-sided unpaired t-test) for the pooled data with corresponding Log_2_-fold change and p-value. D) Biosynthesis of 4-aminobutyric acid.

Since the average significance level was poor for most metabolites when the individual data sets were treated separately, the data was also pooled to give a clearer picture of the effects of the inhibitor. However, since two different cell-lines were used and the GC-MS data was recorded on two different occasions, there were significant variations between the controls in the different data sets. Hence, each metabolite was normalized against the average of the controls in the individual data sets before they were compared. Multivariate differences between the pooled controls and treated samples were examined with PLS-DA (Fig 2A and 2B). Only metabolites detected in > 50% of the samples were included in the model.

According to the PLS-DA analysis the most important metabolites to describe the differences between the controls and the treated samples are aspartic acid, ribose-5-phosphate, and ribose (upregulated in treated samples) and 4-aminobutyric acid, α-ketoglutaric acid, and alanine (downregulated). This is also verified by the student’s t-tests of the pooled data (Fig 2C).

The combination of metabolite data from the two cell-lines and independent replicates improved the significance and strongly support hydrazinosuccinate as an inhibitor of AAT since both aspartic acid and α-ketoglutaric acid were substantially affected. However, there is no evidence that glutamic acid, one of the other substrates, is affected. This indicates that the cells compensate for changes in glutamic acid concentration through other pathways. Neither the enzyme’s cofactor pyridoxal phosphate nor the fourth substrate, oxaloacetate, could be detected in the samples. It is also noteworthy that although aspartic acid and α-ketoglutaric acid are on the same side of the equilibria, one is upregulated and the other downregulated. This illustrates the complexity of how to interpret metabolomics data in terms of drugs mode of actions, especially for multi-substrate enzymes at equilibria.

One of the most clearly affected metabolites was 4-aminobutyric acid. This could indicate an off-target effect on the enzyme glutamate decarboxylase (GAD), a pyridoxal-dependent enzyme that synthesizes 4-aminobutyric acid from glutamic acid (Fig 2D). It is reasonable to believe that this enzyme can be affected by hydrazinosuccinic acid given the structural similarity between the inhibitor and the substrate of the enzyme, and that AAT and GAD operates with similar mechanisms using pyridoxal phosphate as coenzyme. Another metabolite that was reduced by treatment with hydrazinosuccinic acid was alanine. A probable explanation for this is off-target inhibition of alanine aminotransferase, which have previously been demonstrated [21]. Hydrazinosuccinic acid is however more selective than *e*.*g*. the inhibitor AOA, which strongly reduced alanine synthesis in breast cancer cells [19]. The effects of hydrazinosuccinic acid on ribose and ribose-5-phosphate levels are more difficult to explain and further work is needed to determine if this is an indirect effect of the inhibition of AAT or separate off-target effects.

## Conclusions

Hydrazinosuccinic acid, a known inhibitor of the enzyme AAT, proved to affect the intracellular levels of two of the enzyme’s substrates in T-cell lines. The data also suggest off-target effects of at least two more targets, alanine aminotransferase and glutamate decarboxylase. This illustrates how metabolomics data can give important information to help elucidate the mode of action and potential off-target effects of experimental drugs.

## Supporting information

S1 Data Annotated metabolite data. (XLSX)
